# The United Nations Material Assistance to Survivors of Cholera in Haiti: Consulting Survivors and Rebuilding Trust

**DOI:** 10.1371/currents.dis.1b01af244fe3d76d6a7013e2f1e3944d

**Published:** 2017-10-23

**Authors:** Phuong N Pham, Niamh Gibbons, Patrick Vinck

**Affiliations:** Department of Global Health and Population, Harvard T.H. Chan School of Public Health, Harvard University, Cambridge, Massachusetts, United States; Harvard Medical School, Harvard University, Cambridge, Massachusetts, United States; Harvard Humanitarian Initiative, Department of Global Health and Population, Harvard T.H. Chan School of Public Health, Harvard University, Cambridge, Massachusetts, United States; Department of Global Health and Population, Harvard T.H. Chan School of Public Health, Harvard University, Cambridge, Massachusetts, United States; Harvard Medical School, Harvard University, Cambridge, Massachusetts, United States

## Abstract

**Introduction:**

In August 2016, the United Nations (U.N.) Secretary General acknowledged the U.N.’s role in the cholera epidemic that has beset Haiti since 2010. Two months later, the Secretary General issued a historic apology to the Haitian people before the U.N. General Assembly, for the organization’s insufficient response to the cholera outbreak. These steps are part of the U.N.’s “new approach” to cholera in Haiti, which also includes launching a material assistance package for those most affected by cholera.

**Methods:**

This paper draws on the authors’ experience and findings from consultations with more than 60,000 victims and communities affected by disasters and violence in a dozen countries. We reviewed the literature on best practices for consultation with and outreach to communities affected by development and transitional justice programming, and reviewed our own findings from previous studies with a view to identifying recommendations for ensuring that the assistance package reflects the views of people affected by cholera.

**Results:**

The assistance package program is an opportunity to rebuild the relationship between the victims and the United Nations. This can only be achieved if victims are informed and engaged in the process. This consultation effort is also an opportunity to answer a set of key questions related to the nature, structure, and implementation of the victims’ assistance program, but also how the program may be designed to contribute to rebuilding Haitians’ confidence in the U.N. as an institution that promotes peace, human rights, and development.

**Discussion:**

We recommend that the consultations must be accompanied by an outreach effort that provides clear, accurate information on the assistance program, so that it begins to establish a dialogue between the U.N. and cholera victims. Finally, we conclude by offering a number of concrete next steps that the U.N. can take to kick start the consultation process.

## Introduction

In October 2010, ten months after an earthquake killed over 200,000 people and displaced another one million, the first cholera outbreak in Haiti in at least a century was officially announced. By January 2017, approximately 805,000 cholera cases had been recorded by the Haitian Ministry of Public Health and Population (MSPP), 9,483 of whom had died.^1^ The epidemic had its most profound impact during the first year, with an estimated 490,000 cases and nearly 7,000 deaths.^2^ Recent figures indicate that it has abated, but is far from eradicated: there were over 1,856 new suspected cases and 28 new deaths in January 2017 alone.^1^ Several studies have found that the actual toll could be higher than official MSPP numbers suggest,^3^^,^^4^^,^^5^ and there was an upsurge in cases caused by Hurricane Matthew’s damages to the county’s water and sanitation infrastructure in Southern Haiti in October 2016.^6^

Studies have also found that unsafe sanitation arrangements at the United Nations Stabilization Mission in Haiti (MINUSTAH) peacekeeping base at Mirebelais resulted in the introduction of contaminated feces into the Meille River.^7^ Mirebelais is an arrondissement in the Centre Department of Haiti and the Meille is a tributary of Haiti’s longest river, the Artibonite, an important water source for populations living nearby. The bacteria spread extremely rapidly due to a variety of factors, including the wide scope of agricultural and household use of contaminated water from the Artibonite River (e.g., for drinking, bathing and washing); generally poor water and sanitation conditions in the country; the population’s lack of immunity; and people seeking treatment in facilities that were poorly equipped to contain infection.^8^ Some have also attributed the rapid spread to poor decision-making among humanitarian actors after the earthquake.^9^^,^^10^

In August 2016, the United Nations (U.N.) Secretary General acknowledged the U.N.’s role in the cholera epidemic. Two months later, then-Secretary General Ban Ki Moon issued a historic apology to the Haitian people before the U.N. General Assembly, for the organization’s insufficient response to the cholera outbreak. These steps are part of the U.N.’s “New Approach” to cholera in Haiti, which also includes launching a “Material Assistance” package for those most affected by cholera. In March 2017, the current Secretary General reaffirmed the U.N.’s commitment to the New Approach, but the details of its implementation remain in doubt, held up by lack of funding commitments from U.N. Member States to the U.N. Haiti Cholera Response Multi-Partner Trust Fund.^11^^,^^12^

This paper begins with a discussion of how the Material Assistance package can contribute to rebuilding Haitians’ confidence in the U.N. as an institution that protects human rights, and the role that community consultations can play. We then address three main considerations for an effective consultation process: First, who should be consulted? Second, how should they be consulted? And third, what should be asked?

## Methods

This paper builds on the authors’ experience interviewing over 60,000 randomly selected adults in a dozen countries affected by war and complex emergencies on their perceptions of justice and accountability, including reparations as a form of transitional justice (TJ).^13^ We reviewed the literature on best practices for consultation with and outreach to communities affected by development and transitional justice programming, and reviewed our own findings from previous studies with a view to identifying recommendations for ensuring that the assistance package reflects the views of people affected by cholera. No personal identifying information was collected from respondents in most of the studies, and when personal information was gathered, it was removed prior to analysis. The data used for this study were fully anonymized before any of the authors of this study had access to the data.

**1. Material Assistance, Accountability, and Reparations**

As far as accountability is concerned, our hypothesis is that the Haitian population sees the cholera epidemic as one element of a long history of un-redressed human rights violations. In 2016, Haiti ranked 159th of 176 countries in Transparency International’s corruption index – an improvement from 2003, when it was the 3rd country from the bottom of the index.^14^^,^^15^ The widespread human rights abuses under the governments of both Francois and Jean-Claude Duvalier – from 1957–1986 – are well-documented, but since Jean-Claude Duvalier died in 2015 during an investigation targeting him for crimes against humanity, political instability has held up the investigation of his subordinates.^16^ Haitians economic and social rights have suffered during decades of poor governance and were further eroded by the 2010 earthquake's destruction of health infrastructure and an estimated 250,000 homes. Recent reports suggest that hundreds of thousands of people are still living in temporary accommodations.^17^

Looking at the U.N.’s role, activists for justice for cholera survivors note that the cholera epidemic is associated in the public eye with broader perceptions of misconduct by - and impunity for - personnel of MINUSTAH, the peacekeeping mission operational in Haiti since 2004. The U.N. has recorded 107 separate allegations of sexual exploitation and abuse by MINUSTAH personnel, mostly military and police, from 2007 to 2016, and the true number of sexual abuse incidents may be significantly higher.^18^ Some particularly egregious incidents have incited anger among Haitians, and although some misconduct cases have been investigated by the troop-contributing countries or by the U.N., reports suggest that there has been little accountability and no reparation to victims.^19^ These and other scandals have eroded the public’s support for the U.N. presence in Haiti.^20^

Against this context, the Material Assistance package for survivors of cholera is an opportunity for the U.N. and Member States to “get it right for victims and survivors”, by designing a package that is responsive to their needs and views. Notably, the U.N. does not see Material Assistance as a form of reparation, as this would imply a level of legal liability that the organization denies. It is instead considered a demonstration of moral responsibility, but the U.N. still wants to communicate to Haitians that the assistance is from the organization and is linked to its acceptance of moral responsibility. The assistance package therefore shares some of the goals of reparations, like providing acknowledgement, demonstrating accountability (even if not legal liability), mending relationships, and the restoration of trust. Consultations in advance of the assistance package can begin to address these issues, but while the U.N. continues to call upon Member States to fund the New Approach, any national-level consultations are on hold. One initial “symbolic” project will be implemented in the Mirebelais region in the first half of 2017, which will include limited consultations with key stakeholders and beneficiaries.

Our research among disaster and conflict affected populations builds on practices of local participatory decision-making in the broader international development field, where engaging communities is by now considered essential to ensuring appropriateness, effectiveness, and sustainability in assistance projects.^21^^,^^22^ But consultations related to reparations serve a more unique purpose. A 2010 evaluation of the International Criminal Court’s (ICC) Trust Fund for Victims (TFV) found a strong correlation between victims who knew that the assistance was coming from the ICC, and victims who had a positive attitude toward the ICC as an institution that provides justice. The TFV’s report found that “Reparation is more than an award. It is a process that requires proper, two way communication to fully realize its potential."^23^ This suggests, first, that communicating with communities about the source and purpose of the assistance is essential to linking reparations with accountability. Second, it implies that communications should both provide information and gather the communities’ opinions. Ideally, these would be gathered both before and after the assistance is provided so changes in attitudes can be assessed.

There are important differences between a public health outbreak and a situation of mass violence, particularly in the types and motivations of the harms suffered. The difference in context might mean that the processes by which attitudes change (for example, the restoration of trust) might be different. We do not think, however, that the process of consulting communities should change, beyond being adapted to the local context. Consulting Haitians about material assistance for cholera survivors might in fact be more straightforward than similar assessments in places where formerly warring parties still hold power and influence. And as we have described above, the Haitian population has experienced decades of civil and political rights violations, as well as a consistent failure to ensure their economic and social rights, exacerbated by recurring natural disasters. Providing accountability for the full range of violations is not the U.N.’s responsibility, but the Material Assistance package is one initial step that is within the organization’s control.

**2. Who should be consulted?**

In Haiti, the cholera outbreak has had broad reaching effects on Haitian society, with at least 9% of Haiti’s population having been ill with cholera since 2010. Risk of infection, and strain on the health system, have touched the population as a whole, especially considering that only an estimated 60% of Haitians have access to clean water and only 20% use improved sanitation facilities.^24^ There are therefore many categories of survivors: those who have themselves been ill, who have lost or cared for an ill household member or relative, or who were indirectly affected by the epidemic. Furthermore, the universe of Haitians who consider themselves a victim – whether of corruption, human rights abuses, or earthquake damage – likely captures a large portion of the population. During consultations, a narrow definition of “victims” or “survivors” may create tensions among those who are not recognized as victims and subsequently do not receive assistance. The Material Assistance package will be limited to those affected by cholera, and it is unclear whether any individual awards will be made. Consultations provide an opportunity to assess how the population would respond to different forms of assistance: Would non-cholera sufferers support individual awards for those who lost the most due to illness? Would this be a source of tension or conflict? Do particular social constituencies – based on gender, youth, or occupation in agriculture or otherwise – have different effects from the cholera epidemic? These questions could be addressed to communities themselves and could inform decision-making on the design of the package.

Particular attention should be given to the regions that have been most affected by the cholera epidemic in order to ensure that those most affected by the disease have their views heard. Recent data from the Ministry for Public Health and Population (MSPP) indicate that the highest overall numbers of cases and fatalities since 2010 have been in Port-au-Prince, Artibonite, Centre, and Nord departments.^25^ The consultations should also, however, take account of the proportion of the population that has had cholera, which would involve obtaining overall population data for each department. These data were unavailable at the time of writing this paper but are crucial in order to determine the exposure rate in each region.

**3. How should they be consulted?**

Our experience suggests that there are three key conditions that must be met in order for consultations with victims of violence to be meaningful. First, the consultations must be systematic and broadly inclusive, so that they do not give voice to the opinions of some groups over others. Second, the consultations must be seen as legitimate and impartial, and must include follow-up dialogue with communities to discuss decisions made as a result of the consultations. Finally the consultation must be conducted in a transparent and sensitive manner that allows survivors to speak openly and comfortably about their views. This means that sensitivity to cultural norms and social dynamics is essential, and may require ensuring confidentiality and anonymity. In the same line, the consultation process must involve steps to reduce and, where possible, avoid retraumatization of those being consulted.^26^ These considerations are just as relevant to the context of the cholera outbreak in Haiti as they are for consulting survivors of violence.

Given the complex history of the U.N.’s role in Haiti, the assistance package should include a strong, locally-based outreach strategy. Best practices from the outreach strategies employed by TJ processes and international organizations could be used. In the Central African Republic, our research found that the ICC’s outreach strategy there – which, at that time, primarily utilized mass media, NGO partnerships, and informational meetings – was effective in increasing understanding about the Court among more elite members of society but left behind an ‘information poor’ group who had limited access to media or ability to participate in events.^27^ The consultations prior to the launch of the assistance package are an opportunity to gather data on the population’s access to and trust in various forms of media and information, which can be instrumental in designing a locally-driven outreach strategy.

The budget for outreach should also be considered during the establishment of the assistance package. Lessons can be drawn here too from existing TJ processes: the Colombian Victims Unit has a large budget (over US $19 million) for interaction with victims in 2016, and its activities include training and education campaigns; public round-table events; and continuous data collection on damages suffered by communities.^28^ In contrast, the ICC has a proposed budget of approximately $240,000 per country for outreach in 2017.^29^ Its activities are necessarily more limited than in Colombia, but they still include providing information through outreach events and gathering feedback from large numbers of victims.^30^ An in-depth dialogue with outreach experts would help the U.N. to identify the strengths, challenges, and resources needed for information dissemination and implementation of effective dialogue strategies.

Underlying many of the considerations outlined above is the methodological rigor of the consultation process. An independent, scientifically valid and rigorous approach is more likely to be seen as legitimate and impartial. Local knowledge in this context is essential, but there is value in obtaining outsiders’ perspective and arguably neutrality in relation to the questions at hand. Regardless of who conducts the consultation, thorough documentation of narratives and data provided by participants in a systematic and transparent manner is crucial. Ethical research practices must be observed, such as seeking consent and outlining what, if anything, will be done with the information gathered.

In practice, our assessments rely on mixed qualitative and quantitative methods, incorporating focus groups and/or in-depth interviews along with population-based surveys. Qualitative data provide nuanced discussion about what kind of reparations are needed, rationale, and other contextual information while quantitative data provide comparison statistics on preferences related to the assistance program among victims from various geographic locations. The research approach can be rolled out in tandem with an existing program to assess population-level indicators of program effectiveness or to inform program design.^31^ Random, multi-stage sampling from the adult population in the most affected geographic areas would generate data from direct survivors, while a national sample would assess the general public’s views for comparison. In addition, the survey combined with exposure rates can inform the scale, scope, and resources needed for the assistance program.

As a complement to a nationwide outreach and dialogue process, the assistance package could benefit from undertaking deliberative polling, for example, based on the model developed by the Center for Deliberative Democracy at Stanford University. This approach is specifically designed for conducting public opinion research on “issues where the public may have little knowledge or information, or where the public may have failed to confront the trade-offs applying to public policy."^32^ The process involves inviting a sample of several hundred participants to a central location during which they are polled on their opinions on an issue; then provided with detailed, objective information on the advantages, challenges, and trade-offs surrounding the issue; and then polled again after discussion of the information. This approach could be undertaken on a limited, cost-effective basis in Haiti: even with a small sample size, a set of deliberative focus groups could yield important insights.

**4. What should be asked?**

A natural starting point to consult survivors of the cholera epidemic in Haiti would be to examine the various forms of harm (physical, psychological, economic…) and the needs of those affected. Limiting the consultation to a needs assessment, however, would fail to recognize the importance of shaping an assistance program that is seen as legitimate, recognized as a form of recognition of the U.N.’s role, and contributes to the restoration of trust. In a 2015 study in three countries on resilience in peacebuilding settings, we found that social cohesion, leadership and governance, and addressing the past are key factors affecting resilience.^33^ For these reasons, the consultation process should seek to identify a set of key questions related to the implementation of the Material Assistance program and how it will contribute to rebuilding Haitians’ confidence in the U.N. as an institution that promotes peace, human rights, and development. These should be refined in consultation with local stakeholders and international experts to ensure they are appropriate to the Haitian context. However, based on our previous consultations and research, some initial groups of questions are identified below.

*Accountability and trust*

The Material Assistance must be perceived as distinct from other forms of development assistance and humanitarian aid in the country, if it is to be seen as a measure of recognition of the U.N.’s role in the epidemic, and contribute to rebuilding relations between the U.N. and the population. Our research in Colombia found that, when victims who received reparations were asked what purpose the reparations served, the most common response was recognition by the state (53%), followed by recognition of their rights (51%), and building confidence in the state (37%).^34^ For these reasons, the consultation should engage the population on questions relating to perceptions of accountability and trust, and the degree to which reparations will build trust. Key questions may include:

- How does the population view the issue of accountability for the cholera outbreak?

- How is the establishment of the assistance package being received? Has it improved the population’s confidence in the U.N.? In other institutions?

- What form of assistance would survivors most value? Is the goal of the assistance to provide restitution, compensation, rehabilitation, and/or satisfaction, four of the forms of reparation outlined in the 2005 U.N. “Basic Principles and Guidelines on the Right to a Remedy and Reparation?"^35^

- Given the ongoing cholera outbreak, is it possible to provide a guarantee of non-recurrence (the fifth form of reparations identified in the U.N. Guidelines)? Is the population aware of the U.N.’s intensified efforts to improve Haiti’s water and sanitation infrastructure as part of the New Approach?

*Priorities and needs*

Understanding the priorities and needs of those affected is a core component of the consultation process. It is essential, however to use an open-ended approach to assess the many forms of harms, priorities and needs. The key questions may be relatively simple:

- What are the different types of losses and harm?

- What are the most pressing needs?

But these can yield significant insight about the various patterns of victimization and the resulting needs, especially when examined across various social groups (gender and economic lenses for example). What our previous studies show is that there is a wide range of potential themes in terms of victims’ priorities, losses, and needs, and that these can change rapidly over time. Victims frequently emphasize financial compensation but it is not the only acceptable form of assistance and reparation.

*Forms of assistance*

Relating to the needs as outlined by victims themselves, it is important to note that self-identified needs and priorities may not be realistic or feasible. The consultation process must also examine the acceptability of various forms of reparations and directly assess the value of symbolic measures and assistance provided on a group or community basis, as well as to individuals. Eligibility can also be discussed. Equal awards can send a message that one experience of the illness does not have a higher value than another, however it can also be a source of resentment if the needs and economic status of survivors are not adequately considered.^36^ Less differentiation also creates a more streamlined process: defining and compensating based on different classes of victims can place an overwhelming economic and administrative burden on reparations programs. Key questions include:

- Who should be eligible for assistance?

- Should the assistance be provided equally to all individuals affected by cholera? If not, how should assistance be allocated? Based on harm? Based on needs and inability to recover? Based on other criteria?

- Is symbolic and or/collective assistance sufficient under some conditions? If yes, when? Should all reparations be individually-based, community-based, or a combination of both?

Community-based assistance is often seen as acceptable, but these issues, based on our previous studies, are highly contextual. Acceptance of various forms of assistance stems not only from individual preferences, but also from the population’s understanding of what is feasible in their own context.

*Institutional arrangements*

Importantly, in a context of limited trust in institutions, the assistance program must be designed in a manner that builds confidence among the population. For example, it will require a registration process that is seen as unbiased, free of corruption, and with a high level of public confidence in the actors in charge of providing assistance. For these reasons, it is important to engage survivors in defining the institutional arrangements that are most likely to be seen as effective and trustworthy. Key questions may include:

- What kind of process should be used to assess losses and needs?

- What institution would the population trust to implement the program? What institution would be trusted to provide oversight?

- What challenges or barriers might face vulnerable groups – including women, children, the elderly and disabled, those living in remote areas, or those who lack formal documentation of their losses – in accessing the assistance program?

Many reparations programs, including those in Colombia and Cote d’Ivoire, involve a victims’ registration system, while others, including Uruguay, Nepal, and the Philippines, assess eligibility through a special state body established for that purpose. The institution implementing the program could be within a national ministry – such as the Colombian Victims Unit – or a dedicated office, such as the reparations Directorate established within a broader National Commission for Social Action in Sierra Leone.^37^ International organizations have also implemented reparations, such as the 1991 Compensation Commission created to implement reparations to victims of crimes committed during the Iraqi invasion of Kuwait.^38^ The main question to be explored in this regard is which institution the population trusts to implement the program fairly and transparently.

Our studies have frequently explored populations’ levels of trust in the various institutions such as U.N., NGOs and national systems such as the central government and judicial bodies. One finding is that trust in central government institutions is often low, but local actors are not necessarily trusted either. A stakeholder mapping – which institutions are known to survivors, which are accessible to them, and which are trusted – may help identify those most trusted actors and best channels for an assistance program.

## Conclusion

A consultation process to inform the Material Assistance program for survivors of the cholera epidemic would offer the best chance to “get it right for victims and survivors” and design a program that is responsive to survivors’ needs and priorities while achieving broader goals of recognition of harm, mending relationships, and rebuilding trust. It is an opportunity for the U.N. to counter the perception that MINUSTAH has acted with impunity on Haitian territory. Importantly, this consultation must be seen as only the first step toward a meaningful engagement with communities. Lessons learned in engaging communities about assistance and reparation programs in other contexts offer a way forward for such a consultation. The U.N. should take advantage of the full range of tools at its disposal, employing innovative strategies, best practices, and the expertise that exists globally in communicating and connecting with survivors. This paper briefly addressed key questions that a consultation process must consider: who to consult, how to consult them, and what to ask. Our experience shows that methodologically rigorous consultation processes create a strong basis for dialogue and the design of effective assistance programs. U.N. Member States need to provide financial support for the New Approach, and in the meanwhile, the U.N. can think creatively about maximizing the impact of projects launched under the New Approach by communicating effectively with communities. Moving forward, preliminary steps could include convening a meeting of experts on outreach and consultation with communities affected by violence, disasters, or large-scale public health crises, in order to further delve into the best practices and innovative approaches outlined in this paper. Identifying a credible, independent, impartial entity to conduct the consultations is also important. These steps can be very targeted and cost-effective, but the sooner they begin, the more responsive the assistance package will be to the population’s views.

## Data Availability

All relevant data are available within the paper; this study does not report new data and only quotes from published papers.

## Competing Interests

The authors have declared that no competing interests exist.

## Corresponding Author

Niamh Gibbons: ngibbons@hsph.harvard.edu
